# Four cases of enterovesical fistula and the importance of CT in the diagnosis

**DOI:** 10.1259/bjrcr.20150124

**Published:** 2016-08-31

**Authors:** Shuai Li, Zhipeng Chen, Qian Zhang, Chuanyang Huang, Zhe Wang, Shuqi Du

**Affiliations:** ^1^Department of Urology, Research Institute of China Medical University The First Affiliated Hospital of China Medical University, Shenyang, PR China; ^2^Department of Urology, PLA 202 Hospital, Shenyang, PR China; ^3^Department of Urology, Weifang People's Hospital, Weifang, 261042, Shandong, China

## Abstract

Enterovesical fistula is an abnormal communication between the urinary bladder and intestine. Diverticulitis is the most common aetiology, accounting for approximately 50–70% of cases, and malignancy is the second most common cause, accounting for approximately 20% of cases. However, most patients are hospitalized because of urinary symptoms. The disease can be misdiagnosed if patients have been symptomatic for a long time before the diagnosis is made. Detection of enterovesical fistula and the underlying disease is important. However, the optimal diagnostic methods have not been clarified. CT scan is the most sensitive diagnostic modality, but should be backed up with cystoscopy, cystography, colonoscopy and barium enema.

## Background

Enterovesical fistula is an abnormal communication between the urinary bladder and intestine.^1^ Causes of enterovesical fistula can be numerous, but they have been divided into five main classes by Chen et al:^2^ congenital, traumatic, tumour, inflammatory and others. In Western countries, the main cause is intestinal diverticulitis (50–70%) and almost all the cases are associated with colonic or bladder fistula.^3^ The second most frequent cause in Western countries is malignant tumours (20%), which are located mainly in the intestine. Other associated tumours include bladder, cervical, ovarian and prostate cancers, and non-Hodgkin's lymphoma of the small intestine. The third most prevalent cause is Crohn’s disease (10%), which occurs mainly in the ileum. In China, the most common cause of enterovesical fistula is malignant tumours of the intestine and the most common sites are the sigmoid colon and bladder, because tumours and diverticulitis tend to be found in the sigmoid colon.^4^ “Other causes” include iatrogenic injury; trauma; foreign bodies in the intestinal tract; radiotherapy; chronic appendicitis;^5^ tuberculosis; and syphilis. The male-to-female ratio is 3 : 1. The lower prevalence in females is owing to interposition of the uterus between the bladder and sigmoid colon, but higher rates of both colovesical and colovaginal fistula have been reported in females who have previously undergone a hysterectomy.^6^

The earliest and most common symptom is irritation of the urinary tract, which is recurrent and difficult to cure. Pathognomonic features of pneumaturia and faecaluria appear later. Other possible causes of pneumaturia must be excluded using cystoscopy (*eg* urinary tract infections caused by *Enterobacter aerogenes*). Misdiagnosis as urinary tract infection is possible. Other associated symptoms include fever, abdominal/pelvic pain, haematuria, dysuria, testicular swelling and leakage of urine from the anus. Gastrointestinal symptoms such as constipation, abdominal distention, diarrhoea, obstruction and abdominal pain caused by inflammation are possible. High compliance of the bladder and low intravesical pressure mean that pneumaturia and faecaluria are more common (50–90%) than urine flow into the rectum (15%).^7–8^ Hence, urine cultures usually reveal coliform bacteria such as *Escherichia coli* and *Enterococcus* spp.

## Case reports

A summary of the demographic data and clinical presentation of all the participants is given in Tables 1 and 2.

**Table 1.** Presenting symptoms

Case	Age/gender	Aetiology	Presenting symptoms	Urine culture	Site of causative lesion
1	69/F	Appendicitis/diverticulitis	She underwent appendectomy 3 months before presentation and had symptoms of urodynia, frequent micturition, abdominal pain, cloudy urine, chills, nausea, vomiting and occasional pyrexia	*Escherichia coli* (1 × 10^4^ CFU ml^−1^),and ESBLs positivity	A fistula between the bladder and ileum
2	62/F	Appendicitis	She had symptoms of haematuria, frequent micturition, urge incontinence and dysuria. She urinated 3–4 times during the day and 4–5 times at night. She was not pyrexic, and chronic gastrointestinal disease was excluded.	*E. Coli* (1 × 10^5^ CFU ml^−1^), and ESBL positivity	A fistula in the appendix and bladder
3	59/M	Diverticulitis	He had symptoms of urodynia, urge incontinence, gross haematuria and abdominal pain. At its most severe, he was urinating > 30 times per day. Yellow and black cloudy floccules were visible in the urine.	*Enterococcus faecium* (1 × 10^5^ CFU ml^−1^)	A vesicocolic fistula
4	54/M	Colon cancer	He suffered 2 months of increased urinary frequency, urge incontinence, urodynia, dysuria and haematuria, along with hypogastralgia of 1 month’s duration. Occasionally, he suffered rigors at night.	*E. faecium* (1 × 10^4^ CFU ml^−1^)	A vesicocolic fistula

CFU, colony forming units; ESBL, extended-spectrum β-lactamase; F, female; M, male.

**Table 2.** Investigations and findings

Case number	Diagnostic findings indicative of enterovesical fistula				Cystoscopy	Colonoscopy
	Fistulous tract visualized	Air in bladder	Bladder and/or bowel-wall thickening	Extravesical mass that often contained air		
1 (Figure 1)	N	Y	Y	Y	A hemispherical uplift, the centre of which showed mucosal oedema with altered follicles, on the right rear aspect of the bladder wall	Multiple diverticula in the colon
2 (Figure 2)	Y	N	Y	Y	A mass in the bladder with white stones within it (size, 2.5 × 1.5 cm). On the right aspect of the wall was a fistula (diameter, ~1 cm) that was associated with pus and yellow floccules. The fistula bifurcated ~2 cm distally	When the colonoscope was 5 cm from the ileal valve, we injected methylene blue dye into the bladder. The blue liquid was seen to flow out from the appendix
3 (Figure 3)	N	Y	Y	N	A “strawberry-like” tumour (size, 0.3 cm) on the left aspect of the posterior wall of the bladder, surrounded by mucosal folds. Crystallization and precipitation were seen on the fundus of the bladder.	Multiple diverticula in the sigmoid colon (size, 0.2 × 0.2 cm)
4 (Figure 4)	N	N	Y	Y	A substantial mass that appeared to be a tumour (size, 4 × 4 cm) had several floccules adhering to it.	The distance from the circular tumour to the anal margin was 20 cm

Y, yes; N, no.

**Figure 1.**
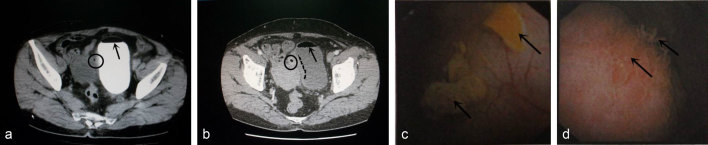
(a) CT scan shows gas (arrowhead) and contrast medium within the bladder, and (b) thickening on the right aspect of the bladder wall (dotted line) with a localized mass (~2.5 × 1.7 cm) and small gas bubbles(circle) were visible. The CT value was 37 Hounsfield units and the enhanced CT value was 72 Hounsfield units. Cystoscopy (c,d)shows bullous oedema and mucosal papillomatous hyperplasia(arrow)

**Figure 2.**
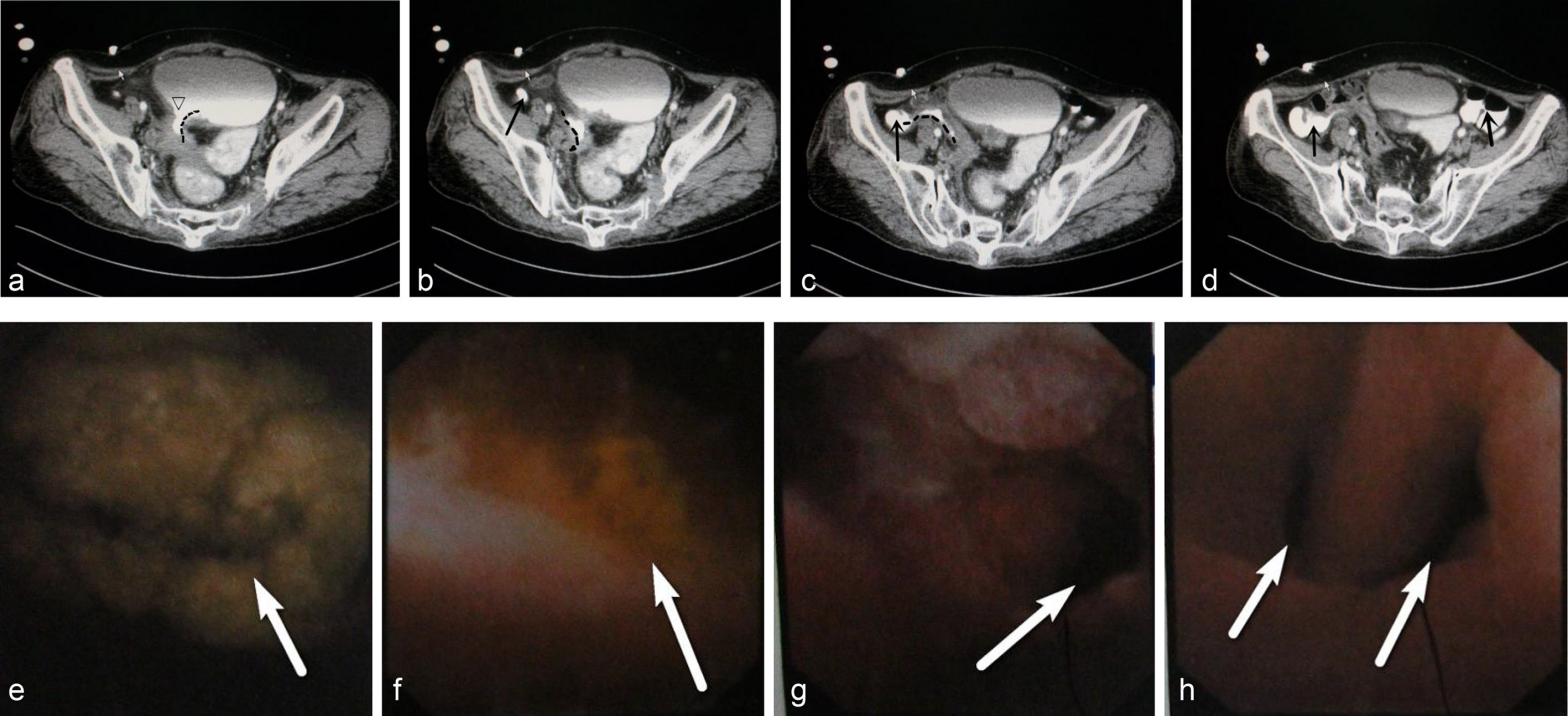
(a, b) CT scan revealed the right aspect of the bladder wall to be thickened (maximum thickness, ~0.97 cm). The fistula (dotted curved line) was visible. On the right posterior wall of the bladder, a discontinuous region of ~1 cm was noted (triangle). (c, d) Contrast agent was observed in the bladder, and part of the intestine (which appeared to be the ileum) was conjoined. Contrast agent filled the caecum (arrowhead). (e) Cystoscopy demonstrated a mass in the bladder with white stones (arrow) within it (size, 2.5 × 1.5 cm). (f) On the right aspect of the wall was a fistula (diameter, ~1 cm) that was associated with pus and yellow floccules (arrow). (g) Opening of the fistula (arrow). (h) The fistula (arrow) bifurcates at ≈2 cm.

**Figure 3.**
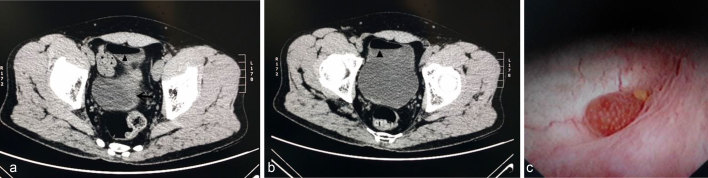
(a) In this case, non-contrast CT scan was performed because of poor health of the patient. (b) The bladder appeared to be thickened, with gas (triangle) visible within it. (c) Cystoscopy demonstrated a “strawberry-like” tumour (size, 0.3 cm) on the left aspect of the posterior wall of the bladder, surrounded by mucosal folds.

**Figure 4.**
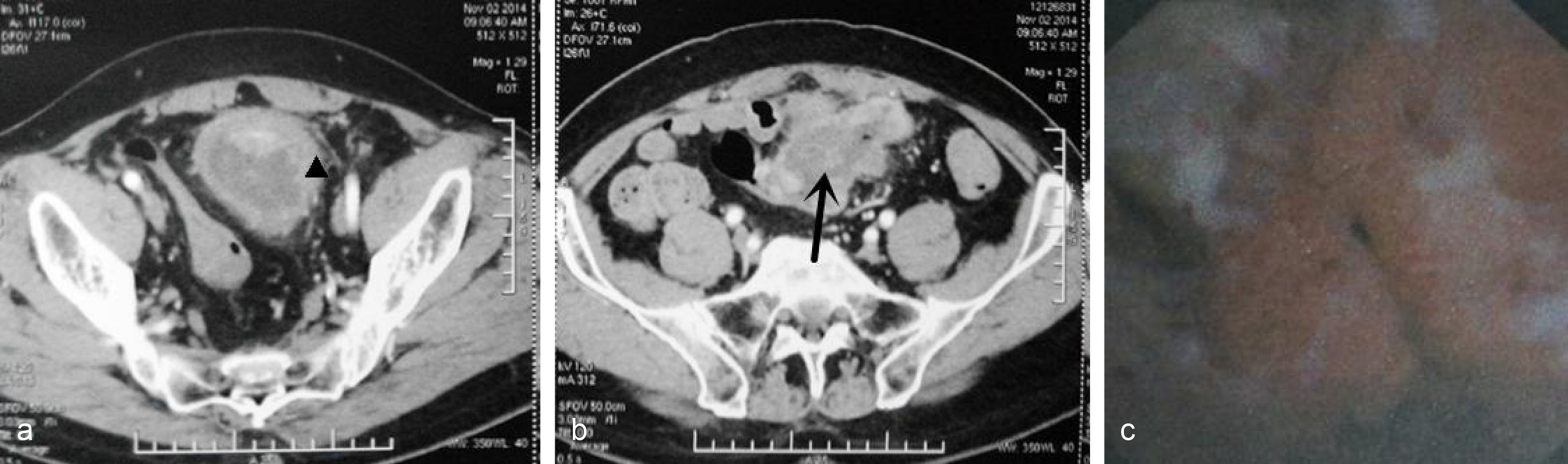
(a) CT scan showed the bladder wall to be irregularly thickened and the serosa was coarsened. (b) Protrusion of soft tissue shadows was visible on the left anterior part of the bladder (size, 4.6 × 6.8 cm). The mean CT value was 30 Hounsfield units, and the enhanced CT value was 67 Hounsfield units. The bladder wall was not smooth but it had multiple enhanced nodules. (c) A substantial mass, which appeared to be a tumour (size, 4 × 4 cm), had several floccules adhering to it.

The four patients were suffering from appendicitis, colon diverticula and colonic carcinoma. All cases were treated surgically. The fistula and part of the bowel were removed, and the bladder wall was repaired.

## Discussion

The traditional diagnostic methods are cystoscopy, barium enemas, colonoscopy and cystography. Barium enemas can be used to locate the primary lesion, but sometimes the contrast agent can flow into the bladder. However, the sensitivity of barium enemas is only 20–35%.^9^ Radiography of centrifuged urine samples obtained immediately after a barium enema (Bourne test) can improve the diagnostic accuracy to 90%, as reported by Gruner et al.^10^ A positive charcoal test is characterized by blackened urine after oral consumption of charcoal, and has ≤ 100% sensitivity for detection of fistulae.^3^ But both the tests provide no information about the fistula’s location or nature.

Simple imaging of the bladder does not help in making a diagnosis. Cystography may demonstrate contrast outside the bladder, but is unlikely to demonstrate a fistula because inflammatory oedema or underfilling of the bladder causes closure of the outlet of the fistula. Cystography shows only gas and elevation of the bladder wall at the site of attachment. However, if there is severe obstruction of the urinary outlet or the patient’s position is changed to increase abdominal pressure, cystography has an increased diagnostic yield. Cystoscopy is considered to be an essential investigation by some authors because it can show fistula and can be used to exclude urological malignancies, bladder stones and interstitial cystitis. A localized area with erythema, oedema and congestion is a typical finding in the early stages of a fistula. Subsequently, bullous oedema and mucosal papillomatous hyperplasia surround a fistula. Floating masses (*eg* faecal residue) can also be seen. However, cystoscopy fails to identify a fistula in > 50% of patients and shows only intravesical oedema, suggesting a fistulous tract, stool and mucus in the bladder. Colonoscopy is not particularly accurate for the detection of fistula. However, colonoscopy is sensitive for the detection of an underlying colonic malignancy, especially colonic malignancies associated with diverticular disease. The use of MRI in diagnosing a fistula has been poorly investigated. It is not clear whether MRI offers any significant benefits over a CT scan and, given that CT imaging is more readily available, an MRI should be used as a second-line investigation for the diagnosis of a colovesical fistula, such as in the imaging of complex fistulae.^9^

The most sensitive and non-invasive examination is a CT scan,^10^ which has a diagnostic accuracy of 60–100%.^3^ Observation of contrast agent in fistulae on enhanced CT scans can help in diagnosing the disease. Detection of a mass outside the bladder wall, local thickening of the bladder wall and thickening of adjacent bowel wall, combined with cystoscopy, can aid in the localization of fistulae. Compared with other methods, the advantage of a CT scan lies in its ability to demonstrate accompanying soft tissue masses forming the fistula between the bladder and the bowel; identify intestinal diverticula; identify adhesions between the intestine and the bladder; show abscess formation; and enable tumour staging for surgical management. The most common findings are gas in the bladder; local thickening of the bladder wall; thickening of adjacent bowel wall; and adherence of soft tissue masses to the outside of the bladder wall.^11,12^ Some authors choose an oral (rather than intravenous) contrast agent to avoid opacification of the bladder.^3,13^ Sometimes, the contrast agent is too viscous to pass through a smaller fistula. The choice of contrast agent and the concentration needed to pass through a fistula have not been studied.

Drug therapy can be curative for some patients with a fistula in the bladder or intestine, but surgery remains the best way to treat the disease. There is no unified surgical approach; it is based on the individual situation. The most common method is to remove the affected bowel and bladder wall forming the fistula, and repair the defect by cystostomy or a urethral catheter. If the disease originates from a malignant tumour, partial or total remodelling of the bladder is required, combined with chemotherapy and regular review.^14^

## Conclusions

CT scan is a useful, sensitive, effective, and non-invasive technique for the evaluation of enteroneovesical fistulae. We recommend that a CT scan should be performed first for a patient with a suspected enterovesical fistula, as it can accurately detect the underlying aetiology of the fistula, it is the most sensitive investigation in demonstrating the fistulous tract, it can identify extraluminal disease, and it can aid in operative planning.However, gastrointestinal endoscopy should be performed to evaluate the colon lumen and cystoscopy should be performed if there is suspicion of underlying bladder cancer.

CT scan is the most sensitive diagnostic modality, but should be backed up with cystoscopy, cystography, colonoscopy and barium enema.

## Learning points

1. Enterovesical fistula is an abnormal communication between the bladder and intestine.
2. Most patients are hospitalized because of urinary symptoms. The pathognomonic features are pneumaturia and faecaluria.
3. The traditional diagnostic methods, including cystoscopy, barium enemas, colonoscopy and cystography, have low sensitivity.
4. CT scan is the most sensitive diagnostic modality and should be performed first for a patient with suspected enterovesical fistula.
5. A significant advantage of CT scan over other techniques is its unique ability to identify extraluminal disease and aid in operative planning.

## Consent

Written informed consent for the case to be published (including images, case history and data) was obtained from the patient(s) for publication of this case report.
